# Exposure to Benzo[a]pyrene Decreases Noradrenergic and Serotonergic Axons in Hippocampus of Mouse Brain

**DOI:** 10.3390/ijms24129895

**Published:** 2023-06-08

**Authors:** Walaa Slouma Hamouda Abd El Naby, Cai Zong, Alzahraa Fergany, Frederick Adams Ekuban, Saleh Ahmed, Yousra Reda, Harue Sato, Sahoko Ichihara, Natsuko Kubota, Shinya Yanagita, Gaku Ichihara

**Affiliations:** 1 Department of Occupational and Environmental Health, Faculty of Pharmaceutical Sciences, Tokyo University of Science, Noda 278-8510, Japan; walaa.slouma@yahoo.com (W.S.H.A.E.N.); zongcai@rs.tus.ac.jp (C.Z.); zhraa.fergany@alexu.edu.eg (A.F.); frederick.ekuban@louisville.edu (F.A.E.); saleh.ahmed.bge@gmail.com (S.A.); yousrar3@gmail.com (Y.R.); 3b16037@alumni.tus.ac.jp (H.S.); 2 Genetics and Genetic Engineering in Department of Animal Husbandry and Animal Wealth Development, Faculty of Veterinary Medicine, Alexandria University, Alexandria 21500, Egypt; 3 Division of Gastroenterology, Hepatology, and Nutrition, Department of Medicine, Pharmacology and Toxicology, University of Louisville, Louisville, KY 40202, USA; 4 Department of Environmental and Preventive Medicine, Jichi Medical University, Shimotsuke 329-0498, Japan; saho@jichi.ac.jp; 5 Faculty of Science and Technology, Tokyo University of Science, Noda 278-8510, Japan; nkubota0824@gmail.com (N.K.); yanashin@rs.tus.ac.jp (S.Y.)

**Keywords:** benzo[a]pyrene, neurotoxicity, noradrenergic axons, serotonergic axons, genes expression

## Abstract

Epidemiological studies showed the association between air pollution and dementia. A soluble fraction of particulate matters including polycyclic aromatic hydrocarbons (PAHs) is suspected to be involved with the adverse effects of air pollution on the central nervous system of humans. It is also reported that exposure to benzopyrene (B[a]P), which is one of the PAHs, caused deterioration of neurobehavioral performance in workers. The present study investigated the effect of B[a]P on noradrenergic and serotonergic axons in mouse brains. In total, 48 wild-type male mice (10 weeks of age) were allocated into 4 groups and exposed to B[a]P at 0, 2.88, 8.67 or 26.00 µg/mice, which is approximately equivalent to 0.12, 0.37 and 1.12 mg/kg bw, respectively, by pharyngeal aspiration once/week for 4 weeks. The density of noradrenergic and serotonergic axons was evaluated by immunohistochemistry in the hippocampal CA1 and CA3 areas. Exposure to B[a]P at 2.88 µg/mice or more decreased the density of noradrenergic or serotonergic axons in the CA1 area and the density of noradrenergic axons in the CA3 area in the hippocampus of mice. Furthermore, exposure to B[a]P dose-dependently upregulated *Tnfα* at 8.67 µg/mice or more, as well as upregulating *Il-1β* at 26 µg/mice, *Il-18* at 2.88 and 26 µg/mice and *Nlrp3* at 2.88 µg/mice. The results demonstrate that exposure to B[a]P induces degeneration of noradrenergic or serotonergic axons and suggest the involvement of proinflammatory or inflammation-related genes with B[a]P-induced neurodegeneration.

## 1. Introduction

Millions of individuals are exposed to air pollution daily. As a result, air pollution-related morbidity and mortality continue to be significant public health concerns around the world. One of these air pollutants is benzo[a]pyrene (B[a]P), the most characterized family member of polycyclic aromatic hydrocarbons (PAHs), due to its carcinogenic effect on different organs in humans and experimental animals. B[a]P is released into a variety of environments including air, water, or soil from both natural and man-made sources, such as industrial pollution, automobile exhaust fumes and cigarette smoke [1,2,3]. Epidemiological studies showed that neurobehavioral functions, learning ability and autonomic nervous function were negatively associated with the airborne B[a]P level in coke oven workers [4,5,6,7]. Experimental studies showed that exposure of rats or mice to B[a]P by intraperitoneal injection caused learning and memory deficits, an anxiolytic-like behavioral response and induced loss in dendritic length and number in the hippocampus [8,9,10,11,12]. Exposure of adult male rats to B[a]P by intraperitoneal injection induced an anxiolytic-like behavioral response, an increase in glutathione S-transferase activity and the concentration of oxidized glutathione, and a decrease in reduced glutathione, glutathione peroxidase activity, glutathione reductase activity, total dendritic length and total dendritic density, suggesting the involvement of oxidative stress with the neurotoxicity of B[a]P [8]. Exposure of rats to B[a]P induced suppression of motor activity and inhibition in the activity of superoxide dismutase and glutathione peroxidase as well as an enhancement in catalase and lipid peroxidation in the striatum and hippocampus [13]. Exposure of 4-month-old APPSwe-PS1DeltaE9 (APP/PS1) mice and wild type mice to B[a]P at 1 mg/kg bw for 2 months induced a progressive decline in spatial learning/memory and exploratory behavior, the activation of microglia and astrocyte and the upregulation of inducible nitric oxide synthase, glial fibrillary acidic protein (GFAP) and nicotinamide adenine dinucleotide phosphate (NADPH) oxidase in the cortex and hippocampus as well as alterations in expressions of cortical genes related to inflammation and the immunoregulatory process, beta amyloid (Aβ) secretion and degradation and synaptic formation. Subacute exposure of adult mice to B[a]P at 0.02 or 0.2 mg/kg bw impaired short-term learning and spatial memory performance in a Y maze and in the Morris water maze tests [9]. Subacute exposure to B[a]P also modulated gene expression of the N-methyl-D-aspartate receptor 1 subunit (NMDA-R1) in brain areas including the hippocampus involved in cognitive functions. Despite the above studies suggesting the involvement of oxidative stress, inflammatory response and NMDA-R1 with the neurotoxicity of B[a]P, the mechanism of B[a]P-induced neurotoxicity remains elusive.

The present study investigated the effect of intratracheal exposure to B[a]P, which simulates air pollution exposure, focusing on noradrenergic axons involved in cognitive functions [14] and serotonergic axons involved in the anxiety phenotype [15] in the hippocampus of mice. In addition, the study investigated the possible role of oxidative stress and inflammatory response by gene expression analysis. 

## 2. Results

### 2.1. Body and Brain Weight

Exposure to B[a]P for 4 weeks did not affect body weight and brain weight irrespective of the exposure level.

### 2.2. Quantification of Noradrenergic and Serotonergic Axons 

**Figure 1:**
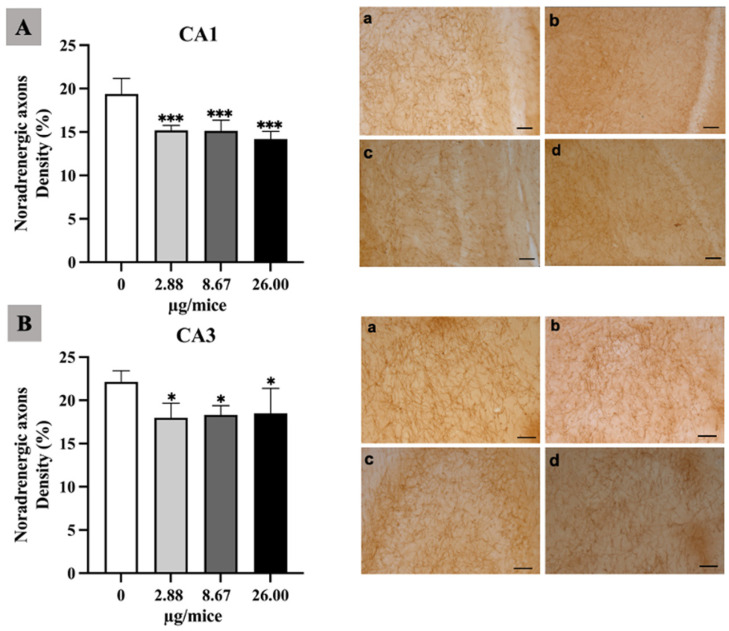
Effect of exposure to B[a]P by pharyngeal aspiration once/week for 4 weeks on the density of noradrenergic axons in hippocampal CA1 (**A**) and CA3 (**B**) area. Control group (**a**), group exposed to 2.88 μg/mice (**b**), 8.67 μg/mice (**c**), and 26.00 μg/mice (**d**), which are approximately equivalent to 0.12, 0.37 and 1.12 mg/kg bw, respectively. Scale bars = 40 μm. Data are mean ± SD. * *p* < 0.05 and *** *p* < 0.001.

**Figure 2:**
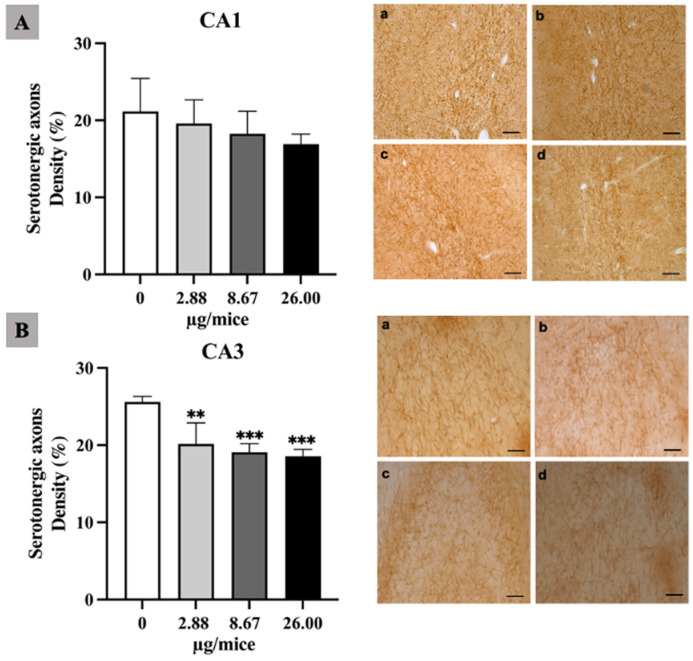
Effect of exposure to B[a]P by pharyngeal aspiration once/week for 4 weeks on the density of serotonergic axons in the hippocampal CA1 (**A**) and CA3 (**B**) area. Control group (**a**), group exposed to B[a]P at 2.88 μg/mice (**b**), 8.67 μg/mice (**c**), and 26.00 μg/mice (**d**), which are approximately equivalent to 0.12, 0.37 and 1.12 mg/kg bw, respectively. Scale bars = 40 μm. Data are mean ± SD and Statistical significance: ** *p* < 0.01 and *** *p* < 0.001.

As the hippocampus plays a crucial role in regulation of learning, memory encoding, memory consolidation and spatial navigation, the density and length of noradrenergic and serotonergic axons was quantified in the hippocampal *cornu ammonis* (CA1 and CA3) regions. The density of noradrenergic axons was significantly reduced in the hippocampal CA1 and CA3 area for all B[a]P treated groups (Figure 1A,B). Moreover, B[a]P exposure induced a significant reduction in the density of serotonergic axons in the CA3 hippocampal area at 2.88 µg/mice or more compared with the control as indicated in Figure 2A,B. 

### 2.3. Histopathological Changes in Lung and Brain Tissue

**Figure 3:**
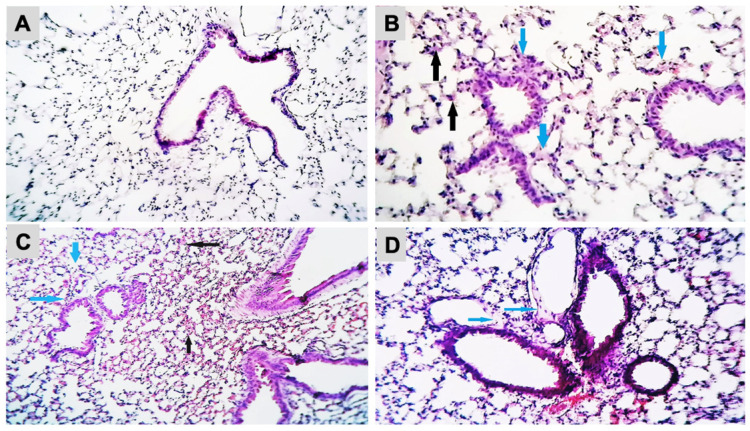
Histopathological changes in mice lung tissue after exposure to B[a]P by pharyngeal aspiration, once/week for 4 weeks. (**A**) Control group, (**B**) 2.88 μg/mice (0.12 mg/kg bw) group showed thickening of the alveolar epithelium (black arrows) with infiltration of inflammatory cells and interstitial edema (blue arrows) (H&E, X400), (**C**) 8.67 μg/mice (0.37 mg/kg bw) group showed the presence of blood (black arrows) and inflammatory cells in interstitial tissue (blue arrows) (H&E, X100) and (**D**) 26 μg/mice (1.12 mg/kb bw) group showed the severe destruction of the alveolar epithelium and the presence of alveolar edema (blue arrows) (H&E, X250).

The histopathological analysis of the lung sections of the control and experimental groups was shown (Figure 3A–D). Exposure to B[a]P at 2.88 µg/mice induced a thickening of the alveolar epithelium with infiltration of inflammatory cells and interstitial edema. The presence of blood and inflammatory cells in interstitial tissue were observed in the mice exposed to B[a]P at 8.67 µg/mice. Mice exposed to B[a]P at 26 µg/mice showed severe destruction of the alveolar epithelium and the presence of alveolar edema. The histopathological analysis of the brain did not show neuronal death or degeneration in the hippocampus either in the control or B[a]P-treated groups (Supplementary Figure S1).

### 2.4. The Expression Levels of Selected mRNA

**Figure 4:**
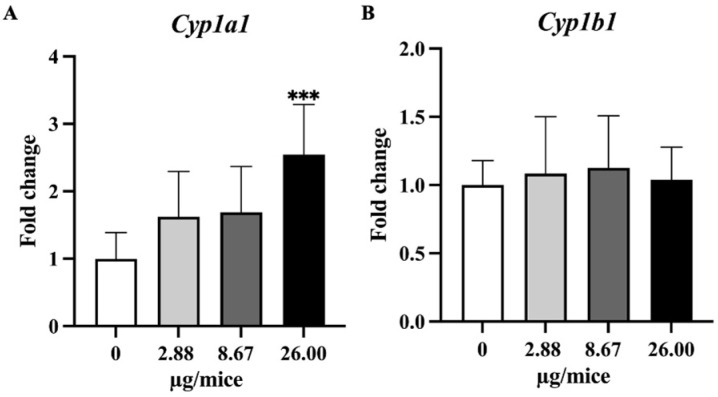
Relative hippocampal expression levels genes involve in B[a]P metabolism. (**A**) *Cyp1a1* and (**B**) *Cyp1b1*. Mice were exposed to B[a]P by pharyngeal aspiration once/week for 4 weeks at 0, 2.88, 8.67 and 26.00 µg/mice, which are approximately equivalent to 0.12, 0.37 and 1.12 mg/kg bw, respectively. Data were presented as the mean ± SD and statistical significance: *** *p* < 0.001.

**Figure 5:**
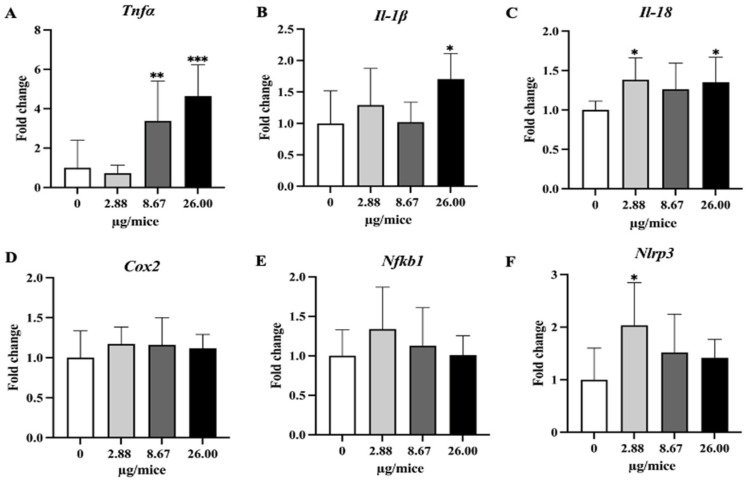
Relative hippocampal expression levels of pro-inflammatory cytokines and the inflammatory mediator. (**A**) *Tnfα*, (**B**) *Il-1β*, (**C**) *Il-18*, (**D**) *Cox2*, (**E**) *Nfkb1* and (**F**) *Nlrp3*. Mice were exposed to B[a]P by pharyngeal aspiration once/week for 4 weeks at 0, 2.88, 8.67 and 26.00 µg/mice, which are approximately equivalent to 0.12, 0.37 and 1.12 mg/kg bw, respectively. Data were presented as the mean ± SD and statistical significance: * *p* < 0.05, ** *p* < 0.01, and *** *p* < 0.001.

**Figure 6:**
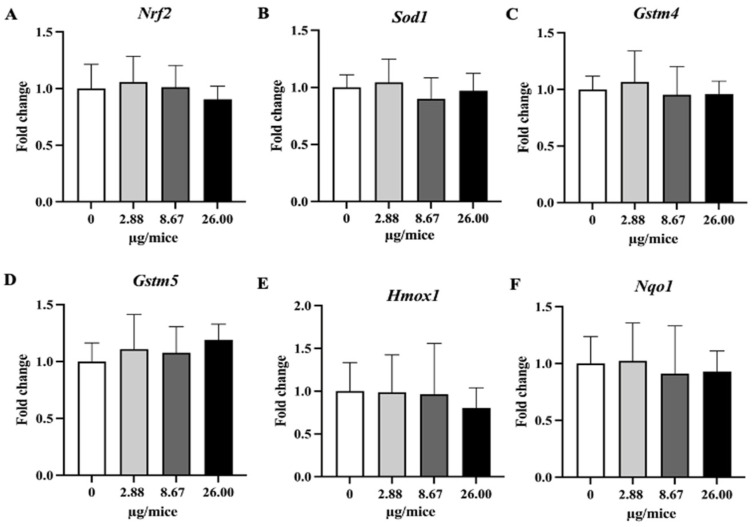
Relative hippocampal expression levels of *Nrf2* and its downstream antioxidant genes. Mice were exposed to B[a]P by pharyngeal aspiration once/week for 4 weeks at 0, 2.88, 8.67 and 26.00 µg/mice, which are approximately equivalent to 0.12, 0.37 and 1.12 mg/kg bw, respectively. (**A**) *Nrf2*, (**B**) *Sod1*, (**C**) *Homx1*, (**D**) *Nqo1*, (**E**) *Gstm4* and (**F**) *Gstm5* mRNAs. Data were presented as the mean ± SD.

**Figure 7:**
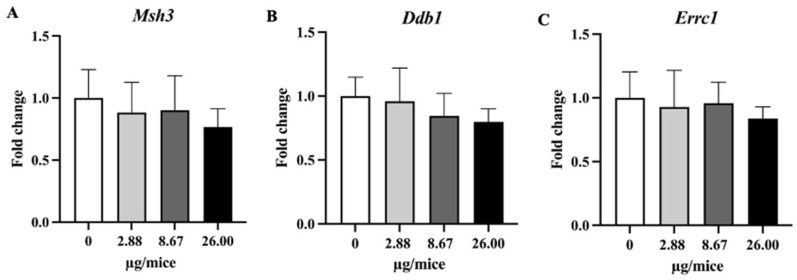
Relative hippocampal expression levels genes involved in the DNA repair process. (**A**) *Msh3*, (**B**) *Ddb1* and (**C**) *Ercc1* genes. Mice were exposed to B[a]P by pharyngeal aspiration, once/week for four weeks at 0, 2.88, 8.67 and 26.00 µg/mice, which are approximately equivalent to 0.12, 0.37 and 1.12 mg/kg bw, respectively. Data were presented as the mean ± SD.

In the current study, the effect of B[a]P exposure on the expression of genes involved in B[a]P metabolism, pro-inflammatory or inflammatory mediator genes, *Nrf2* and its downstream antioxidant genes and genes involved in the DNA repair process in mouse hippocampus tissue was quantified using quantitative real-time PCR (Figure 4, Figure 5, Figure 6 and Figure 7).

As *Cyp1a1* and *Cyp1b1* genes are essential and play a crucial role in the oxidation and metabolism of B[a]P to its metabolite, expression levels of *Cyp1a1* and *Cyp1b1* genes were analyzed. Exposure to B[a]P dose-dependently induced *Cyp1a1* mRNA expression with a significant change at 26 µg/mice in hippocampus tissue (Figure 4A,B). Exposure to B[a]P significantly upregulated *Tnfα* at 8.67 and 26 µg/mice, *IL-1β* at 26 µg/mice, *Il-18* at 2.88 and 26 µg/mice and *Nlrp3* at 2.88 µg/mice (Figure 5A–C,F). 

B[a]P exposure did not change expression levels of *Nrf2*, the examined antioxidant genes and the DNA repair gene (*Msh3*, *Ddb1*, and *Ercc1*) in hippocampal tissue (Figure 6 and Figure 7).

## 3. Discussion

The present study demonstrated that exposure to B[a]P reduced the density of noradrenergic axons in CA1 and CA3 and serotonergic axons in CA3 in the hippocampus of mice. Exposure to B[a]P upregulated gene expression of *Cyp1a1*, *Tnfα*, *Il-1β* and *Il-18* largely in a dose-dependent manner and that of *Nlrp3* only at a low exposure level, while it did not change the expression of the examined antioxidative genes. The study reveals the adverse effect of B[a]P on noradrenergic and serotonergic axons in the hippocampus of mice and suggests the involvement of inflammatory pathways with the neurotoxicity of B[a]P.

A previous study using Golgi staining showed that the exposure of adult rats to B[a]P induced a loss in total dendritic length and number with reduced spin density in the hippocampus, but it did not specify the type of affected axons [8]. Our study revealed that exposure to B[a]P decreased noradrenergic and serotonergic axons in the CA1 or CA3 region of the hippocampus, and noradrenergic axons were more susceptible to B[a]P exposure than serotonergic axons in the CA1 region of the hippocampus in mice. 

The hippocampus is known to play a pivotal role in learning and memory processes [16,17]. In Alzheimer’s disease, there is a significant loss of *locus coeruleus* noradrenergic neurons projecting to the hippocampus [14]. On the other hand, decreased serotonergic activity has been implicated with anxiety and major depression [15]. The result on noradrenergic axons should be noted as epidemiological studies show a negative association between B[a]P exposure and learning ability [4,5,6,7] and experimental studies show that exposure to B[a]P induces learning and memory impairment [8,9,10,11,12].

The greater susceptibility of noradrenergic axons than serotonergic axons in the CA1 region might be related to a previous study which shows that the exposure of female rats to B[a]P by intraperitoneal injection at 50 mg/kg bw decreased the level of noradrenaline (NA), dopamine (DA) or 3,4-dihydroxyphenylacetic acid (DOPAC) after 24 h, but did not induce a major alteration in 5-hydroxytryptamine (5-HT) or 5-hydroxyindoleacetic acid (5-HIAA) in the striatum. NA decreased in the hypothalamus after 96 h and the most important change in midbrain was a decrease in NA after 24 h [18]. Our previous study demonstrated that exposure to 1-bromopropane by inhalation decreased the density of noradrenergic axons in the dorsal medial prefrontal cortex, ventral medial prefrontal cortex and basolateral nucleus of the amygdala of rat brains but did not change the density of serotonergic axons in the same brain regions [19], and exposure to acrylamide through drinking water decreased the density of noradrenergic axons in the dorsal medial prefrontal cortex of mouse brains to a greater extent than it decreased the density of serotonergic axons in the same brain region [20]. As metabolism of benzopyrene leads to the production of electrophilic metabolites [21,22] and 1-bromopropoane or acrylamide belongs to the group of electrophiles, there might be a common mechanism for greater susceptibility of noradrenergic axons than serotonergic axons to electrophiles.

Exposure to B[a]P induced lung inflammation including thickening of the epithelium with infiltration of inflammatory cells and interstitial edema at low concentrations and presence of blood and inflammatory cell in interstitial tissue, destruction of the epithelium or alveolar edema at higher concentrations, thus we may raise the possibility that hypoxia may induce neuronal damage. However, this is unlikely because most alveoli were pneumatic and no abnormality in breathing or arousal level was observed throughout the experiment, and in addition hematoxylin eosin staining did not show any neuronal cell death in the hippocampus CA1 region (Supplementary Figure S1), which is observed in neuronal damage from acute hypoxia [23].

In the present study, exposure to B[a]P upregulated the expression of proinflammatory cytokines (*Tnfα*, *Il-1β* and *Il-18*) and *Nlrp3*. A previous study showed that exposure to B[a]P at 1mg/kg bw by intraperitoneal injection for 2 months upregulated the gene expression of *Tnfα*, but downregulated that of *IL-1β* in wild-type mice [24]. It is possible that the level of gene expression of *Il-1β* depends on the duration or level of exposure. In this regard, B[a]P-induced production of *IL-1β* is demonstrated by an in vitro study that exposure of mouse microglia BV2 cells to B[a]P at 10 µM resulted in the production of pro-inflammatory factors, including nitric oxide, *IL-1β*, and *IL-6, iNOS* and *COX-2* [25]. Another study demonstrated that exposure of mice to B[a]P at 10 and 50 mg/kg bw, once per day for 7 days by trachea injection increased the protein expression of cyclooxygenases-2 (COX-2) in the cortex and exposure of primary cortical neurons to a metabolite of B[a]P, benzopyrene diol epoxide (BPDE) increased the COX-2 level [26]. Neuronal insults were reduced by COX-2, EP-2, EP-4 or PKA blockers in vitro *and in vivo*, suggesting the role of COX-2 and its cascade pathway in B[a]P-induced neuronal damage. As COX-2 and downstream effector molecules play a pivotal role in inflammation, the study demonstrates the role of inflammation in the neurotoxicity of B[a]P.

On the other hand, the expression of Nrf2-dependent antioxidant genes in the hippocampus was not affected by B[a]P exposure in the present study. A previous study [13] showed that exposure to B[a]P by gavage at 25 mg/kg bw or more inhibited the activity of superoxide dismutase and glutathione peroxidase, and increased catalase activity and lipid peroxidation, suggesting the production of oxidative stress, but it should be noted that the exposure level was far higher compared to the present study. The present study shows that exposure to B[a]P upregulates the expression of proinflammatory cytokine genes but does not upregulate antioxidative genes, suggesting a greater role of the inflammatory pathway than oxidative stress in B[a]P-induced neurodegeneration at low exposure level.

After B[a]P enters the body, it binds with aryl hydrocarbon receptor (AhR) and enhances the expression of Cytochrome P450 genes which metabolize the B[a]P into benzo(a)pyrene-7,8-epoxides that are further hydrated into diol epoxides, which are very active metabolites, by epoxide hydrolase [27,28,29]. In the present study, the *Cyp1a1* expression level was significantly upregulated at 26 µg/mice, while *Cyp1b1* did not show large alterations in its expression. The brain is one of the extrahepatic tissues that express CYP1A1 both constitutively and following induction [30,31]. In a recent study, exposure to B[a]P by oral gavage at 20 and 200 mg/kg for 11 consecutive days induced significant overexpression of *Cyp1a1/Cyp1b1* in the frontal cortex, temporal cortex, and hippocampus regions of mouse brains [32]. The difference in the result of *Cyp1b1* between the previous study and the present study might be due to the difference in exposure level. 

Exposure to B[a]P once per week for 4 weeks did not affect body weight and brain weight, irrespective of exposure level. The result on body weight is in agreement with a previous study [26] which reported that exposure to B[a]P at 0.02–200 mg/kg/day for 11 days had no effect on body weight. However, the result of brain weight is different from a previous study which showed that exposure to B[a]P by intraperitoneal injection at 3.2 and 7.8 mg/kg bw decreased the brain weight [33]. The discrepancies between the present study and the previous study might be due to differences in exposure level, and duration of treatment.

The present study has limitations. Firstly, although the present study reveals the adverse effect of intratracheal exposure to B[a]P on noradrenergic axons in mouse brain, the study has a limitation as the study does not reveal molecular factors involved in the adverse effect of B[a]P on noradrenergic axons in mouse brains. Secondly, only adult male mice were used. The neurotoxicity of benzopyrene might depend on neurodevelopmental stage or sex.

In conclusion, exposure to B[a]P decreases the density of noradrenergic and serotonergic axons. Upregulation of proinflammatory cytokines may contribute to the neurotoxicity of B[a]P, but further studies are needed to understand fully the mechanism of toxicity. 

## 4. Materials and Methods

### 4.1. Chemicals 

B[a]P was purchased from (A2385, Sigma Aldrich Co., LLC, St. Louis, MO, USA), dissolved in a modified dispersion medium (DM) of 5.5 mM D-glucose and 0.01 mg/l 1,2-dipalmitoyl-sn-glycero-3-phosphocholine (DPPC) in Ca^2+^ and Mg^2+^-free phosphate buffer saline (PBS), sonicated by a bath-type sonicator, and stored in a refrigerator at 4 °C. Bovine serum albumin (BSA) was removed from the original DM, which was developed for the dispersion of nanoparticles and described previously [34,35], to prepare the modified DM to optimize the dispersion of B[a]P.

### 4.2. Animals and B[a]P Administration 

In total, 48 (9 weeks of age and 21–23 gm BWT) wild-type male mice (C57BL/6J) were purchased from CLEA Japan, Inc. (Tokyo, Japan). The mice were kept under standard conditions of temperature (at 21–23 °C) and humidity (47–57%), and in natural light/dark cycles of 12/12 h with food and water ad libitum. All animals were acclimatized for 1 week prior to the initiation of the experiment. The mice were weighed and randomly allocated into 4 groups (*n* = 12/group). After inhaling 5% sevoflurane for 7 min, the mice were exposed to B[a]P by pharyngeal aspiration at 0, 2.88, 8.67 or 26.00 µg/mice once/week for 4 weeks. It was confirmed that anesthesia lasted for one minute after the end of inhalation of sevoflurane. The middle level of 8.67 µg/mice was calculated as the value comparable to the deposited B[a]P in the lungs of mice exposed to B[a]P at 100 µg/m^3^ (recommendation exposure limit for PAH by US NIOSH [36]) by inhalation for 8 h/d for 5 days, given that the tidal volume is 10 mL/kg [37], respiratory rate is 155/min [38], mouse body weight is 23.3 g and all particles in the exchanged air are trapped in the lung. The above B[a]P doses are approximately equivalent to 0.12, 0.37 and 1.12 mg/kg bw. The animals were observed daily, and body weight was measured 2 times/ week. Control group mice inhaled sevoflurane and were treated with the vehicle in the same way as the other treated groups.

All animal procedures were approved (approval number: Y21016) by the animal experiment committee of the Tokyo University of Science. The guidelines of Tokyo University of Science on animal experiments and the Japanese Act on Welfare and Management of Animals were strictly followed throughout the animal experiment. 

### 4.3. Sample Collection 

Four mice from each experimental group were used for histopathological examination. After one week from the last B[a]P exposure, four mice from each experimental group were anesthetized deeply with pentobarbital sodium and perfused via the left ventricle with 4% paraformaldehyde (PFA) solution at 110 mmHg using a pulsatile pump. The whole brains were dissected after leaving the perfused mice on ice for one hour, and postfixed in 4% PFA at 4 °C for an overnight period. The brains were divided into three parts by cutting transversally at the chiasma optics and the border of midbrain and pons, and then dehydrated using different % dilutions of sucrose solutions 10%, 20%, and 30% for an overnight period/each, respectively. Then the right sides of middle part samples were embedded in Tissue-Tek (optimal cutting temperature (OCT)) compound (Sakura Finetek Co., Tokyo, Japan) and frozen on dry ice, then stored at −80 °C until cryosectioning and immunohistochemistry staining. The left sides of the above middle part brain samples and lung tissues collected in 4% PFA were embedded in paraffin for histopathological examination. The remaining eight mice from each experimental group were decapitated and lung and whole brains were removed, and their weight was measured. The whole brain was placed in a mouse brain slicer and cut into different anatomical regions which were immediately frozen on dry ice and stored at −80 °C until biochemical analysis.

### 4.4. Immunohistochemistry for Noradrenergic and Serotonergic Axons 

Noradrenergic and serotonergic axons in the hippocampus were visualized and their densities were quantified following the previous studies [20,39]. Frozen brain blocks were sectioned at 40 μm thickness using a freezing microtome (Leica CM3050S, Leica Microsystems, Wetzlar, Germany). The sections were mounted on Matsunami MAS coated slides (Matsunami Glass Ind. MAS-02, Osaka, Japan) and air-dried for one hour at room temperature then stored at −80 °C until immunostaining. Frozen hippocampus sections were left for 30 min to an hour at room temperature and then hydrated in Tris-buffered saline (TBS; 50 mM Tris, 0.15 mM NaCl, pH 7.5–7.8) for 5 min. Antigen recovery was performed a using preheated Na-Citrate buffer (10 mM, pH 8.5). Sections were placed in a sodium citrate buffer and kept at 80 °C for 30 min, then allowed to cool to room temperature and washed in Tris-buffered saline with 0.01% Tween-20 (TBST). The endogenous peroxidase activity was repressed by incubating the section for 20 min in BLOXALL Endogenous Blocking Solution (Vector Laboratories, Inc., Newark, CA, USA) at room temperature, then sections were washed 3 times with TBST for 5 min/each. The non-specific binding sites were blocked with protein/serum block reagent at 4 °C overnight then subject to continuous blocking for 1 h at room temperature. The biotin sites were blocked by using an Avidin/Biotin blocking kit (Vector laboratories, Inc., USA) in accordance with the directions provided by the manufacturer, then the sections were washed 3 times with TBST for 5 min/each. The sections were incubated with a primary antibody (1:1000 Mouse Anti-Noradrenaline Transporter (NAT) (Abcam, Cambridge, UK) at 37 °C for 2 h or 1:2000 Rabbit Anti-Serotonin Transporter (5-HT) (Immunostar, Hudson, WI, USA) at 37 °C for 48 h), then the sections were washed 3 times with TBST for 5 min/each. Thereafter, the sections were incubated with a secondary antibody (Horse Anti-Mouse IgG antibody (H+L) Biotinylated, R.T.U (BA-2000-50, Vector Laboratories, Inc., Newark, CA, USA) and Biotinylated Horse Anti-Rabbit IgG antibody (H+L) Biotinylated, R.T.U (BP-1100-50, Vector Laboratories, Inc., Newark, CA, USA) for anti-NAT and anti-5-HT, respectively, for 1 h at room temperature. Then, the sections were washed 3 times with TBST for 5 min/each. To allow more accessibility for binding to a biotinylated target, the sections were incubated for 45 min with VECTASTAIN Elite ABC Reagent, Peroxidase (Vector Laboratories, Inc., Newark, CA, USA). The sections were stained by using an ImmPACT^®^ DAB Substrate Kit, Peroxidase (Vector Laboratories, Inc., Newark, CA, USA) as directed by the manufacturer. Finally, the sections were dried and mounted with VectaMount AQ^TM^ Aqueous Mounting Medium (Vector Laboratories, Inc., Newark, CA, USA) then two sections/each animal were visualized with a light microscope (Olympus, Tokyo, Japan). The axon densities in the CA1 and CA3 hippocampus area were quantified in a square frame (300 × 300 μm) from 2 sections/mice in each exposure groups (*n* = 4) by using vessel analysis plugin in Image J software version 1.53t (National Institute of Health, Bethesda, MD, USA) as described in a previous study [20]. 

### 4.5. Conventional Histopathological Analysis of Lung and Brain

Histopathological evaluation was performed on lung specimens fixed in 4% PFA and brain specimens perfused and postfixed in 4% PFA, processed and embedded in paraffin wax. Sections were cut at 4 mm in thickness, stained with hematoxylin and eosin, and examined under a light microscope. 

### 4.6. RNA Isolation, cDNA Synthesis, and Quantitative Real-Time PCR (qPCR) Assay

Total RNA was isolated from entire hippocampus tissue using a ReliaPrep RNA tissue miniprep system (Promega, Madison, WI, USA) according to the manufacturer’s instructions. RNA concentration and purity were determined by using a NanoDrop 2000 spectrophotometer (Thermo Scientific Inc., Waltham, MA, USA). The SuperScript III reverse transcriptase (Invitrogen, Carlsbad, CA, USA) was used for cDNA synthesis according to the manufacturer’s instructions. Briefly, 4 μg of total RNA was mixed with 1 μL oligo (dt) and 1 μL dNTP Mix, 5X first strand buffer, 0.1 DTT, 1 UL RNaseOUT, 1 μL SuperScript III reverse transcriptase, and the reaction was carried out as follows: 65 °C for 5 min, 50 °C for 60 min, 70 °C for 15 min, and 4 °C holds for 1 min. The cDNAs were then stored at 4 °C until analysis. The quantitative real-time PCR was carried out to investigate the expression levels of selected genes by using the THUNDERBIRD^®^ SYBR^®^ qPCR Mix (Toyobo Co., Osaka, Japan) on the AriaMx Real-Time PCR System (Agilent Technologies, Inc., Santa Clara, CA, USA) following the protocol of the manufacturer. The reaction conditions were performed as follows: an initial denaturation step at 95 °C for 1 min, followed by an amplification step of 40 cycles at 95 °C for 15 s, primer annealing at 60 °C for 30 s, extension at 72 °C for 1 min and a melting curve was generated after the last cycle as follows: 95 °C for 30 s, 60 °C for 30 s and 95 °C for 30 s. The primer sequences of selected genes are listed in Table 1.

To quantify the relative expression level of each gene, a standard curve was formed from a serial concentration of diluted cDNA samples from the control group. The relative expression level of each gene was calculated by normalization to the housekeeping gene, glyceraldehyde-3-phosphate dehydrogenase (*Gapdh*) mRNA level.

### 4.7. Statistical Analysis 

The results were analyzed with GraphPad Prism 9 (GraphPad Software 9, La Jolla, CA, USA) and JMP Pro 14.0 also was used to analyze the data (SAS Institute Inc., Cary, NC, USA). The data were presented as the mean ± SD. Significant differences between treatment groups and the control were determined using one-way ANOVA followed by Dunnett’s multiple comparison test. The significance level was set as *p* < 0.05.

## Figures and Tables

**Table 1:** Primers used for quantitative real-time PCR.

Gene Name and Accession No.	Primer Sequence (5′–3′)	Amplicon Size (bp)
*Cyp1a1* NM_001136059	F: ACCCTTACAAGTATTTGGTCGT R: GTCATCATGGTCATAACGTTGG	80
*Cyp1b1* NM_009994	F: TTGACCCCATAGGAAACTGC R: GCTGTCTCTTGGTAGGAGGA	113
*Nfkb1* NM_001410442.1	F: ATTCCGCTATGTGTGTGAAGG R: GTGACCAACTGAACGATAACC	135
*Tnfα* NM_001278601.1	F: CAGGCGGTGCCTATGTCTC R: CGATCACCCCGAAGTTCAGTAG	89
*Cox2* NM_011198.5	F: TTCGGGAGCACAACAGAGT R: TAACCGCTCAGGTGTTGCAC	148
*Il-1β* NM_008361.4	F: TAACCTGCTGGTGTGTGACG R: TGTCGTTGCTTGGTTCTCCT	182
*Il-18* NM_001357222.1	F: TGGTTCCATGCTTTCTGGACTCCT R: TTCCTGGGCCAAGAGGAAGTGATT	132
*Nlrp3* NM_145827.4	F: TCACAACTCGCCCAAGGAGGAA R: AAGAGACCACGGCAGAAGCTAG	147
*Nrf2* NM_010902.5	F: GGACATGGAGCAAGTTTGGC R: TCCAGCGAGGAGATCGATGA	165
*Sod1* NM_011434.2	F: ATTGGCCGTACAATGGTGGT R: ATCCCAATCACTCCACAGGC	116
*Hmox1* NM_010442.2	F: AGGCTAAGACCGCCTTCCT R: TGTGTTCCTCTGTCAGCATCA	72
*Nqo1* NM_008706.5	F: GCAGGATTTGCCTACACATATGC R: AGTGGTGATAGAAGCAAGGTCTTC	81
*Gstm4* NM_001160411.1	F: CTGAAGGTGGAATACTTGGAGC R: GCCCAGGAACTGTGAGAAGA	63
*Gstm5* NM_010360.3	F: AGAAACGGTACATCTGTGGGG R: GGATGGCGTTACTCTGGGTG	141
*Msh3* NM_001311120.2	F: TGCAAGCATACCTACCCACAGA R: CGGCAGTTTCAGTTTGCTTCAC	95
*Ddb1* NM_015735.3	F: TGCAGTGGAGATCTTGGATGATG R: TTGACAAACTCGCCCAGGTG	150
*Ercc1* NM_007948.2	F: CTGGAGACCTACAAGGCGTATG R: GATTTCACGGTGGTCAGACACTC	107
*Gapdh* NM_001289726.2	F: GCCTGGAGAAACCTGCCAA R: TGAAGTCGCAGGAGACAACC	117

## Data Availability

The data presented in this study are available on request from the corresponding author.

## Supplementary Materials

The supporting information can be downloaded at: https://www.mdpi.com/1422-0067/24/12/9895/s1.
