# Supplementary figures and images for: Exposure to Benzo[a]pyrene Decreases Noradrenergic and Serotonergic Axons in Hippocampus of Mouse Brain
Source: Int J Mol Sci. 2023 Jun 8;24(12):9895. doi: 10.3390/ijms24129895 (PMC10297856; doi:10.3390/ijms24129895)

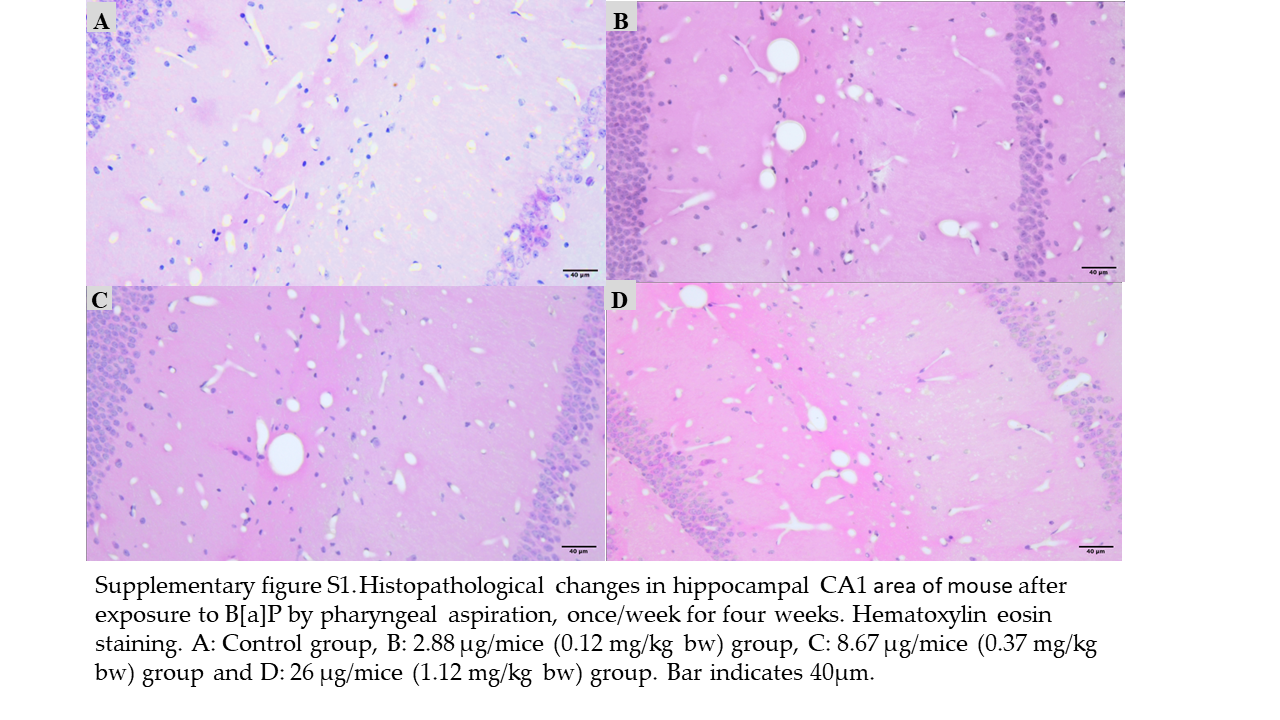

Supplement: Supplementary file 1 [file ijms-24-09895-s001.zip › ijms-2414853-supplementary.tif]
